# RNF38 suppress growth and metastasis via ubiquitination of ACTN4 in nasopharyngeal carcinoma

**DOI:** 10.1186/s12885-022-09641-x

**Published:** 2022-05-15

**Authors:** Cheng Lin, Meifang Li, Na Lin, Jingfeng Zong, Jianji Pan, Yunbin Ye

**Affiliations:** 1 Department of Radiation Oncology, Fujian Medical University Cancer Hospital & Fujian Cancer Hospital, Fuzhou, 350014 China; 2 Department of Medical Oncology, Fujian Medical University Cancer Hospital & Fujian Cancer Hospital, Fuzhou, China; 3 Laboratory of Immuno-Oncology, Fujian Cancer Hospital, Fuzhou, 350014 China; 4 Fujian Provincial Key Laboratory of Translational Cancer Medicine, Fuzhou, China

**Keywords:** Nasopharyngeal carcinoma, RNF38, Growth, Metastasis, Ubiquitination, ACTN4

## Abstract

**Background:**

Accumulated evidence suggests that RING finger proteins (RNFs) are involved in the carcinogenesis of cancers. However, RNF38, a member of the RNF protein family, has not been studied in nasopharyngeal carcinoma (NPC).

**Methods:**

RNF38 expression was analyzed by RT-PCR, Western blotting and Immunohistochemistry. Biological functions of RNF38 were evaluated by cell growth, colony formation, apoptosis, migration and invasion assays in vitro. Xenograft growth and lung metastasis models were conducted to investigate the effect of RNF38 in vivo. Liquid chromatography coupled with tandem mass spectrometry, co-immunoprecipitation, and CHX assay were implemented to detect the interaction among RNF38 and ACTN4.

**Results:**

RNF38 was significantly downregulated in NPC cells and tissues. Immunohistochemistry implied that loss of RNF38 was an independent prognostic factor for poor outcomes of NPC patients. Gain- and loss-of-function experiments showed that RNF38 inhibited proliferation and metastasis in NPC in vitro and in vivo. Upregulation of RNF38 promoted apoptosis of NPC cells to etoposide but not cisplatin. ACTN4 was upregulated in NPC and negatively correlated with RNF38. Mechanistic investigations suggested that RNF38 inactivates the NF-𝛋B and ERK1/2 signaling pathways by inducing ubiquitination and degradation of ACTN4. RNF38 suppress the development of NPC by interacting with ACTN4.

**Conclusions:**

RNF38 plays a potential cancer suppressor gene role in NPC tumorigenesis and is a prognostic biomarker in NPC.

**Supplementary Information:**

The online version contains supplementary material available at 10.1186/s12885-022-09641-x.

## Introduction

Nasopharyngeal carcinoma [1] remains the most common head and neck cancer in southern China [2]. Approximately 70% of patients are diagnosed with locally advanced NPC when first present, and the 5-year overall rate may drop to less than 70%, compared to over 90% in early NPC [3, 4]. To date, with the advantage of intensity-modulated radiation therapy (IMRT), the overall prognosis has significantly improved. However, locoregional recurrence and distant metastasis remain the leading causes of failure in the management of NPC treatment [5]. Therefore, it is necessary to study the exact molecular mechanisms in the pathogenesis of NPC and to find potential prognostic biomarkers and therapeutic targets [6, 7].

The ubiquitin-proteasome system (UPS), including ubiquitin-activating enzymes (E1 s), ubiquitin-conjugating enzymes (E2s), and ubiquitin ligases (E3s), is closely implicated in physiology and pathophysiology [8]. As a major component of E3s, RING fingers are involved in diverse biological processes, such as oncogenesis, signal transduction, apoptosis, development, and viral infection [9]. Currently, accumulating studies have demonstrated that RING fingers (RNFs), such as RNF43 [10], RNF20 [11], RNF125 [12], RNF6 [13, 14], and RNF183 [15], are significantly associated with the development of cancers. Some of these RNF members may be used as novel prognostic biomarkers and potential therapeutic targets in the treatment of cancer. However, as a member of the RNF family, the role of RNF38 (ring finger protein 38) in cancers remains unclear and awaits further identification. RNF38 is mapped to chromosome 9p12–13 [16], a chromosome segment that is frequently deleted in NPC [17] and squamous cell head and neck cancer [18]. It is worth noting that chromosome 9p losses were considered a key early event in the carcinogenesis of NPC [19]. In addition, our team found that RNF38 was a target of the Epstein–Barr virus-encoded microRNA BART8-3p, which promoted metastasis in NPC cells [20]. Moreover, we found that RNF38 was involved in the development of NPC by interacting with ACTN4 according to our preliminary exploration.

ACTN4 (Actinin Alpha 4), one of the actin-binding protein families, is involved in the regulation of cytoskeletal structure and is closely related to epithelial-mesenchymal transition and metastasis of tumor cells [21]. Studies have shown that ACTN4, as an oncogene, plays a role in promoting the development of many cancers [22–26]. The metastatic potential of tumor cells decreases when ACTN4 is downregulated in breast cancer [22]. ACTN4 also participates in the proliferation of tumor cells [25, 26]. Studies in melanoma have shown that ACTN4 promotes growth in melanoma cells by activating the NF-𝛋B signaling pathway, and the transcriptional activation of NF-𝛋B can induce the expression of ACTN4. Therefore, NF-𝛋B and ACTN4 form a positive feedforward loop [26].

To the best of our knowledge, there are no studies to date investigating the function and mechanisms of RNF38 in NPC. Thus, this study aimed to investigate the clinical significance, roles, and mechanisms of RNF38 in NPC. We found that RNF38 acts as a potential cancer suppressor gene in NPC. Patients with lower expression of RNF38 were associated with worse outcomes, and loss of RNF38 was an unfavorable prognostic factor. RNF38 inhibits proliferation, migration, and invasion in vitro and in vivo. Mechanistic investigations suggested that RNF38 inactivates the NF-𝛋B and ERK1/2 signaling pathways by inducing ubiquitination and degradation of ACTN4.

## Materials and methods

### Cell lines and culture

CNE-1 and SUNE-1 cells were purchased from China Center for Type Culture Collection (Wuhan, China). CNE-2 and NP69 were obtained from the Chinese Academy of Science Cell Bank (Shanghai, China). 6-10B was a kindly gift of Prof. Yiwen You from Affiliated Hospital of Nantong University. Four NPC cell lines (CNE1, CNE2, SUNE-1 and 6-10B) were cultured in RPMI medium 1640 (NCS, Hyclone, Invitrogen) supplemented with 10% fetal bovine serum (Gibco, Grand Island, NY, USA). The immortalized normal nasopharyngeal epithelial cell line NP69 was cultured in Keratinocyte-SFM medium supplemented with epidermal growth factor. Cultures were maintained at 37 °C under 5% CO2.

### Patients

Samples from 129 NPC patients and 20 healthy volunteers were collected between January 2007 and December 2009 at Fujian Cancer Hospital. All patients were newly diagnosed and histologically proven with NPC. The TNM stages of NPC were classified according to the International Union Against Cancer/American Joint Committee on Cancer (UICC/AJCC). This study was approved by the ethics committee of Fujian Cancer Hospital (Approved number, SQ2020–056-01).

### Quantitative real-time PCR

Total RNA was extracted using Trizol (Invitrogen) according to the manufacturer’s protocol. To examine the expression of RNF38 mRNA (forward primer: 5′-GCACGCAACAGAAGAAGTCC-3′; reverse primer: 5′-TGGATGAGCAGCAGGATGTAG-3′), complementary DNA (cDNA) was synthesized with the Transcriptor First strand cDNA synthesis Kit (Roche), PCR was performed with LightCycler 480 SYBR green I master (Roche). Data were normalized to GAPDH expression. The fold changes were calculated through the relative quantification method (2^−ΔΔCt^). All reactions were run in triplicate times.

### Lentivirus production and infection

The RNF38 lentiviral vector and control vector were purchased by GeneChem, Shanghai, China. CNE2 cell lines and SUNE1cell lines were stably transfected with RNF38 lentiviral vector (GSV138) or control vector and named RNF38 or NC, respectively. 6-10B cell lines were stably transfected with shRNF38 lentiviral vector (GV248) or control vector and named in-RNF38 or in-NC, respectively. The sequence of shRNF38 was as follows, CTTCCTTCTTATCGGTTCAAT. Enhanced infection solution and polybrene were used to assist the process of lentivirus infecting NPC cells. Puromycin was used to select NPC cells that stably overexpressing and downregulating RNF38. All experiments were carried out on mycoplasma-free cells.

### Western blot analyses

Western blot analyses were performed according to the standard methods. Primary antibodies were listed as follows: anti-RNF38 (1:250, Abcam), anti-ACTN4 (1:1000, Cell Signaling Technology), anti-p-Erk1/2 (1:1000, Cell Signaling Technology), anti-p-p65 (1:1000, Cell Signaling Technology), anti- catenin (1:1000, Cell Signaling Technology), anti-MMP2 (1:200, Cell Signaling Technology), anti-MMP9 (1:250, Abcam). Briefly, the blots were incubated with the following primary antibodies at 4 °C overnight, followed by incubation with the Horseradish Peroxidase-Conjugated secondary anti-rabbit or anti-mouse (1:3000, Santa Cruz) for 1 hour at room temperature. GAPDH (1:10000, Abcam) serves as the endogenous loading control.

### Immunohistochemistry (IHC) examination

Two independent pathologists (Lihua Zhong and Wenhui Liu), who were unaware of the patient characteristics, evaluated the specimens. Expression of RNF38, Ki67, E-cadherin and Vimentin were examined using immunohistochemistry assays by two independent pathologists. The indirect streptavidin-peroxidase method was used according to the manufacturer’s protocol. Anti-RNF38 (1:50, Abcam), Anti-Vimentin (1:200, Cell Signaling Technology), Anti-E-Cadherin (1:400, Cell Signaling Technology), and Anti-Ki67 (1:400, Cell Signaling Technology) were used as primary antibodies. The expression of RNF38 was accessed by the staining intensity and the proportion of positive cells. Testis tissues were used as positive control and NPC tissue stained with the same standards apart from using anti-RNF38 antibody as a negative control. RNF38 was primarily found in the cell nucleus. The staining intensity was graded as 0 (absent), 1 (weak), 2 (mediate), and 3 (strong). The percentage of positive cells was graded as 0 (0%), 1 (< 25%), 2 (25–50%), 3 (50–75%) and 4 (> 75%). The scores were calculated by multiplying the intensity score and percentage score to figure out a semiquantitative H-score. The mean H-score from all patients was used as the cutoff value to determine RNF38 positivity, which was described in our previous article [27].

### Cell proliferation, migration, and invasion assays

Transfected cell lines were seeded in plates at a density of 2000 cells/well and were cultured for 1, 2, and 3 Days. Cell index was calculated by applications named xCELLigence RTCA S16(Real-Time Cell Analyzer, AECA, Biosciences, Inc). The clone assays, cell migration and invasion assays were conducted as described in our previous study [20].

### Apoptosis test

NPC Cells were cultured in six-well plates and treated with 0.01% DMSO, cisplatin (10 um or 20um) or etoposide (80 um) for 48 h. Then, cells were washed twice with cold PBS and resuspended in 1 X Binding Buffer. 100ul of the solution (1× 10^5 cells) was transferred to a new tube, followed by adding 5ul of PE Annexin V and 5ul 7-AAD (PE Annexin V Apoptosis Detection Kit I, BD PharmingenTM), and then incubated for 15 min at room temperature in the dark. Cells were analyzed by Beckman-Coulter system.

### Nude mouse tumor cell xenograft growth and lung metastasis model

Female BALB/c nude mice (4–6 weeks old) were purchased from Shanghai SLAC Laboratory Animal Co., Ltd. (Shanghai, China). All animal studies were performed according to protocols approved by the Institutional Animal Care and Use Committee. Briefly, for the tumor xenograft model, SUNE-1 cells were subcutaneously injected into mouse (2 × 10^7^ cells in 100 μl PBS). After 5 weeks, tumors were removed, photographed, and weighed. Xenografts were monitored daily, and tumor size was measured every four days. For the lung metastasis model, SUNE-1 cells were (1.0 × 10^6^ cells in 100 ul PBS) were intravenously injected through the tail vein. All mice were subjected to fluorescent imaging on ANLT-9MACIMSYSPLUS whole-body imaging system (Lighttools Research; Encinitas, CA, USA) after six weeks. Then mice were sacrificed by CO2 asphyxiation, and pulmonary metastatic lesions were sampled and quantified. Samples of tumor xenografts and lung metastasis model were fixed and paraffin-embedded, and serial 4um sections were conducted for IHC analysis.

### Liquid chromatography coupled with tandem mass spectrometry (LC-MS/MS)

NPC cells with overexpressed Flag-tagged RNF38 was prepared and then anti-Flag antibody was added to pull-down the proteins that may interact with RNF38. Then, 1 mL cold RIPA lysis buffer containing protease inhibitor (Roche) was added to cultured cells to prepare the samples. Next, the mixture was centrifuged 15 min at 4 °C with top speed, and the supernatant was transferred to a new ep and kept on ice. Next, the lysates were incubated with EZview Red ANTI-FLAG M2 Affinity Gel (Sigma-ALDRICH) overnight at 4 °C. Centrifuge for 30 seconds at 8200×g and remove the supernatant carefully. Wash the bead pellet by adding 500 μl of TBS. Vortex, incubate and gentle mixing at 2–8 °C for 5 minutes. Centrifuge for 30 seconds at 8200×g and remove the supernatant. Repeat washing the bead two more times and remove the supernatant. Then, add 50 μL 1% TFA to Dynabeads, incubate 10 min at 37 °C with high-speed shaking to elute binding proteins. Finally, Tryptic digestion and Peptide desalting were conducted for further LC-MS/MS. Raw MS files were processed with MaxQuant (Version 1.5.6.0). This database and its reverse decoy were then searched against by MaxQuant software. Both peptide and protein FDR should be less than 0.01. Only unique & razor peptides were used for quantification.

### Co-immunoprecipitation, ubiquitination assay and CHX assay

For Co-immunoprecipitation assay, cell lysate of SUNE-1-NC and SUNE-1-RNF38 cells were incubated with primary antibody RNF38, ACTN4 or IgG at 4 °C overnight. The mixture was added to protein A/G magnetic beads (Thermo Scientific™ Pierce™), then placed on a low-speed rotating shaker for 1 hour at room temperature. After washed by RIPA buffer three times, proteins were eluted with separated by SDS-PAGE, and immunoblotted with antibodies against RNF38, ACTN4 and GAPDH, respectively. As to ubiquitination assay, SUNE-1-NC and SUNE-1-RNF38 cells were treated with MG132, followed by immunoblotting (IB) analysis to detect ubiquitin. CHX chase assay was used to examine the half-life period of ACTN4, SUNE-1-NC and SUNE-1-RNF38 cells were treated with CHX for 0, 2 and 4 hours, then IB was performed.

### Statistical analysis

Data are analyzed using software SPSS (v24.0; Inc.; Chicago, IL, USA) and Graph Pad Prism 8 (GraphPad; La Jolla, CA, USA). Two sets of data are analyzed using Student’s t-test. The relationship between RNF38 expression and various Characteristics is analyzed by the chi-square test. Kaplan-Meier method and the log-rank test are used to evaluate the survival probability or to determine statistical significance according to RNF38 expression, respectively. Multivariate analysis is performed using the Cox regression model. The correlation between RNF38 and ACTN4 mRNA expression was evaluated with Spearman’s correlation analysis. All statistical tests are two-sided. A *P* value of < 0.05 is considered statistically significant.

## Results

### RNF38 is downregulated in NPC and serves as an independent, favorable prognostic biomarker

**Fig. 1:**
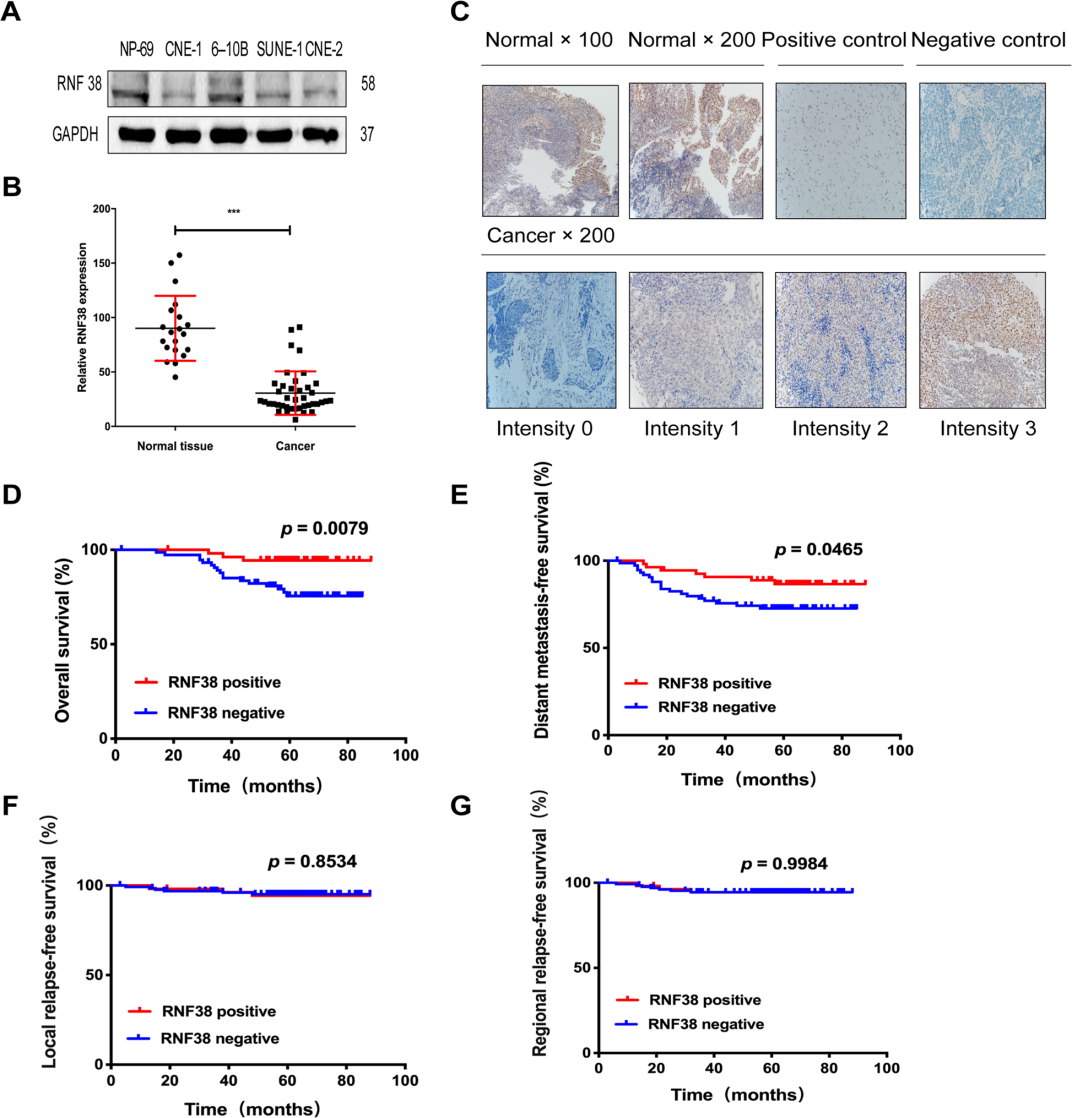
RNF38 expression in nasopharyngeal carcinoma [1] and its clinical significance in NPC patients. **A** RNF38 expression in NPC cells (CNE-1, 6-10B, SUNE-1 and CNE-2) and NP69 cells by Western blot. **B** RNF38 expression in NPC and normal nasopharyngeal tissues by RT-qPCR. **C** Representative images of immunohistochemical staining (IHC) of RNF38 in normal nasopharyngeal tissues, positive and negative controls (Upper column). IHC of RNF38 in NPC by different intensities (Lower column). **D**-**G** Kaplan-Meier survival curves of overall survival (OS), distant metastasis-free survival (DMFS), local relapse-free survival (LRFS) and regional relapse-free survival (RRFS) in 129 NPC patients stratified by RNF38 expression

To explore the function of RNF38 in NPC progression, we first examined RNF38 expression in NPC cell lines (CNE1, SUNE1, CNE-2, 6-10B) and the immortalized normal nasopharyngeal epithelial cell line NP69. The Western blot results showed that NP69 showed the most abundant RNF38 expression, while RNF38 was obviously reduced in the four NPC cell lines (Fig. 1A). This finding was also true for mRNA expression in 20 normal nasopharyngeal and 40 NPC tissues, which was in line with our previous data [20] (Fig. 1B).

To further address the expression and potential prognostic value of RNF38 in patients with NPC, we performed immunohistochemistry (IHC) in 129 NPC and 20 normal nasopharyngeal samples (Fig. 1C). Compared to NPC tissues, RNF38 was significantly overexpressed in healthy nasopharyngeal tissues, with an 80% RNF38-positive rate (Fig. 1C). Of the 129 NPC patients, 54 (41.86%) patients had RNF38-positive tumors (Tables S1 and S2). The expression of RNF38 had no significant differences with clinical characteristics, including sex, age, T classification, N classification, AJCC stage and chemotherapy (*p* > 0.05, Table S3).

Kaplan-Meier analysis revealed that RNF38-positive patients had significantly improved overall survival (OS) (Fig. 1D) and distant metastasis-free survival (DMFS) (Fig. 1E) compared with RNF38-negative patients. There was no significant correlation between RNF38 expression, local relapse-free survival (LRFS) (Fig. 1F) or regional relapse-free survival (RRFS) (Fig. 1G). Multivariate survival analyses confirmed that RNF38 expression was an independent, favorable prognostic factor for DMFS (HR, 0.43; 95% CI, 0.16 to 0.97; *p* = 0.042) and OS (HR, 0.21; 95% CI, 0.06 to 0.73; *p* = 0.010) (Table 1). Collectively, these results indicate that RNF38 may be a favorable potential biomarker for NPC.

**Table 1:** Multivariate survival analysis of risk factors for nasopharyngeal carcinoma patients

Variables	DMFS		OS	
	HR(95%CI)	*p*	HR(95%CI)	*p*
Sex		0.231		0.267
Male vs Female	0.52 (0.17–1.52)		0.49 (0.14–1.72)	
Age at diagnosis		0.214		0.025*
≤ 50 vs > 50	1.65 (0.75–3.65)		2.97 (1.15–7.72)	
T classification		0.375		0.312
T1–2 vs T3–4	0.56 (0.16–2.00)		0.43 (0.09–2.19)	
N classification		0.744		0.374
N0–1 vs N2–3	0.84 (0.29–2.41)		0.55 (0.15–2.05)	
AJCC stage		0.195		0.269
I-II vs III-IV	3.06 (0.56–16.65)		3.09 (0.42–22.77)	
Chemotherapy		0.300		0.519
No vs Yes	2.25 (0.49–18.36)		1.55 (0.41–5.84)	
RNF38 status		0.042*		0.010*
negative vs positive	0.43 (0.16–0.97)		0.19 (0.06–0.67)	

Abbreviations: *OS* overall survival; *DMFS* distant metastasis-free survival; *HR* hazard ratio; *CI* confidence interval; *AJCC* American Joint Committee on Cancer *p < 0.05 was considered significant

### RNF38 inhibits NPC cell proliferation in vitro and in vivo

**Fig. 2:**
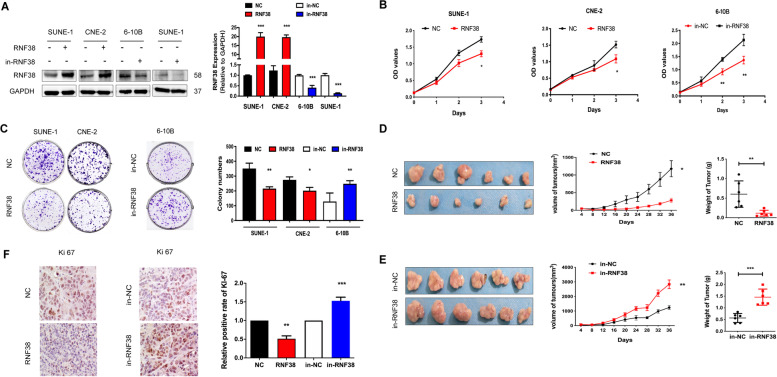
RNF38 inhibits the proliferation of NPC cells in vitro and in vivo. **A** Overexpression of RNF38 (RNF38), downregulation of RNF38 (in-RNF38), and the related normal controls (NC or in-NC) in NPC cells verified by Western blot and RT-PCR. **B-C** Cell proliferation assays and clone formation assays. **D-E** Subcutaneous injection of SUNE-1 cells stably overexpressing or downregulating RNF38 in the xenograft model (*n* = 6). Xenograft tumor volume and tumor weight were analysed. **F** Representative images and quantification of the expression of Ki67 in xenograft tumors. Data are presented as the mean ± SEM; *P* values were calculated using Student’s t-test. *p < 0.05, ** *p* < 0.01, *** *p* < 0.001

To investigate the roles of RNF38 in NPC cells, we conducted upregulating (RNF38) or downregulating (in-RNF38) RNF38 expression cell lines using lentivirus production. As the expression of RNF38 was the highest in 6-10B cells and lower in SENU1 and CNE2 cells, we chose 6-10B cells to down-regulate RNF38 expression and chose SENU1 and CNE2 cells to up-regulate RNF38 expression. The transfection efficiency was confirmed by Western blot and PCR (Fig. 2A). Cellular proliferation assays (Fig. 2B) and colony formation assays (Fig. 2C) suggested that upregulation of RNF38 significantly inhibited NPC cell growth compared with controls. Then, NPC cell proliferation was examined in vivo by subcutaneously inoculating transfected SUNE-1 cells into nude mice. We found that the average xenograft tumor volume and tumor weight was significantly decreased in RNF38 overexpressing cells (RNF38) compared with control cells (NC) (Fig. 2D), and vice versa (Fig. 2E). Additionally, we observed higher Ki67 expression in the RNF38 upregulated group and lower Ki67 expression in RNF38 downregulated group (Fig. 2F). Taken together, these results showed that RNF38 exerted an inhibitory effect on growth NPC.

### RNF38 is involved in the DNA damage response and promotes apoptosis

**Fig. 3:**
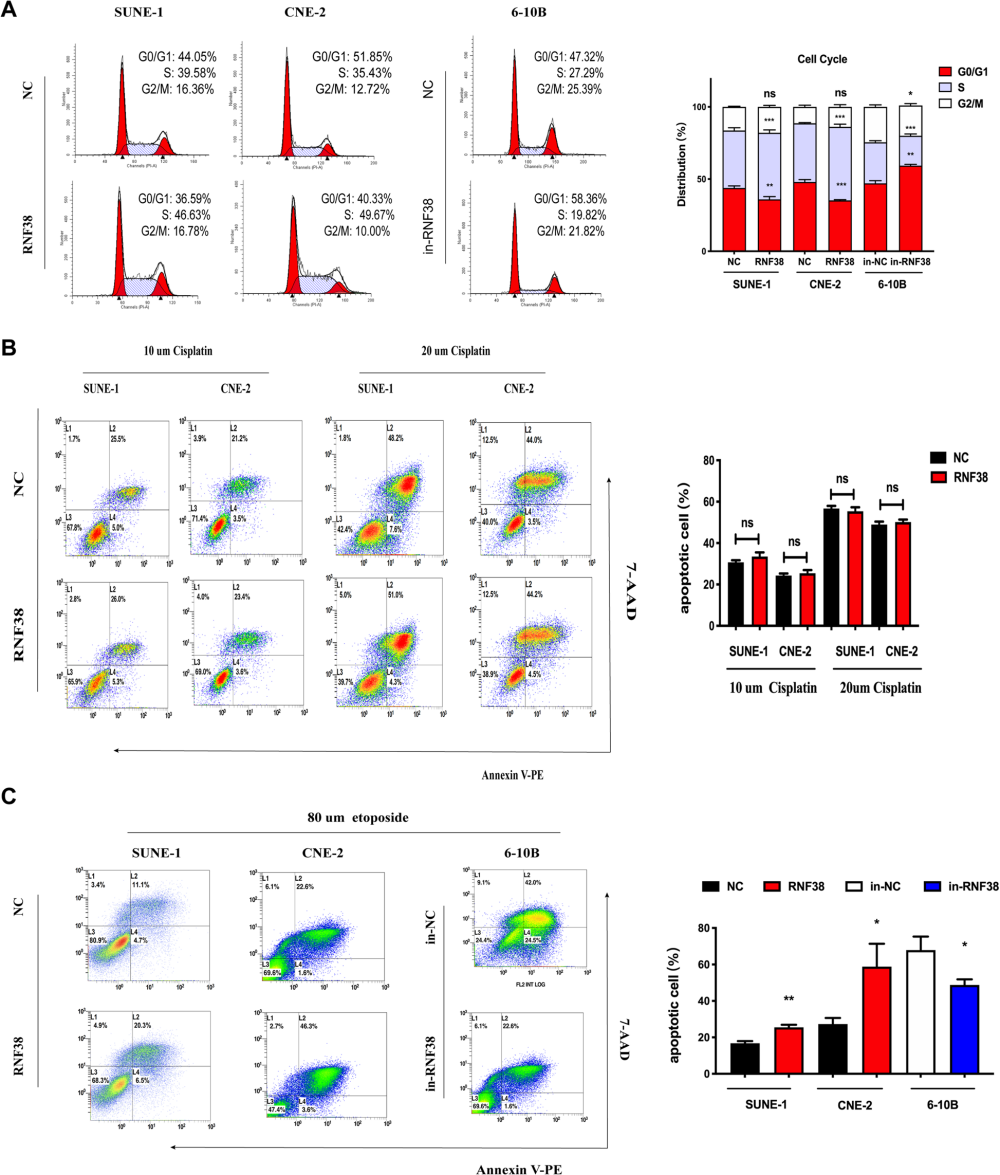
RNF38 affects NPC cell cycle distribution and sensitivity to DNA-damaging drugs. **A** Representative images of flow cytometry and quantification of cell cycle distribution of the cell cycle assays. **B-C** Representative images of flow cytometry and quantification of apoptotic NPC cells treated with cisplatin (Upper column) and etoposide (Lower column). Ns, no significant, *p < 0.05, ** *p* < 0.01, *** *p* < 0.001

Since RNF38 is closely associated with cell proliferation, we wondered how RNF38 affects DNA synthesis in NPC cells. The cell cycle analysis showed that overexpression of RNF38 remarkably arrested the cell cycle in S phase in SUNE-1 and CNE-2 cells (Fig. 3A). In contrast, knocking down RNF38 dramatically increased the proliferation of 6-10B cells and stimulated the transformation of cells from S phase to other phases. Cisplatin, a drug commonly used in NPC therapy, can kill cancer cells by damaging DNA. Our data revealed that compared to control cells, overexpression of RNF38 had no impact on NPC cell sensitivity to cisplatin, regardless of whether the concentration of cisplatin was 10 or 20 μm (Fig. 3B). Interestingly, when treated with etoposide, another DNA-damaging drug, increased NPC cell apoptosis was observed in the RNF38-overexpressing group (Fig. 3C). Therefore, we concluded that RNF38 may play a key role in the apoptosis of NPC cells to etoposide rather than cisplatin.

### RNF38 inhibits NPC cell metastasis in vitro *and* in vivo

**Fig. 4:**
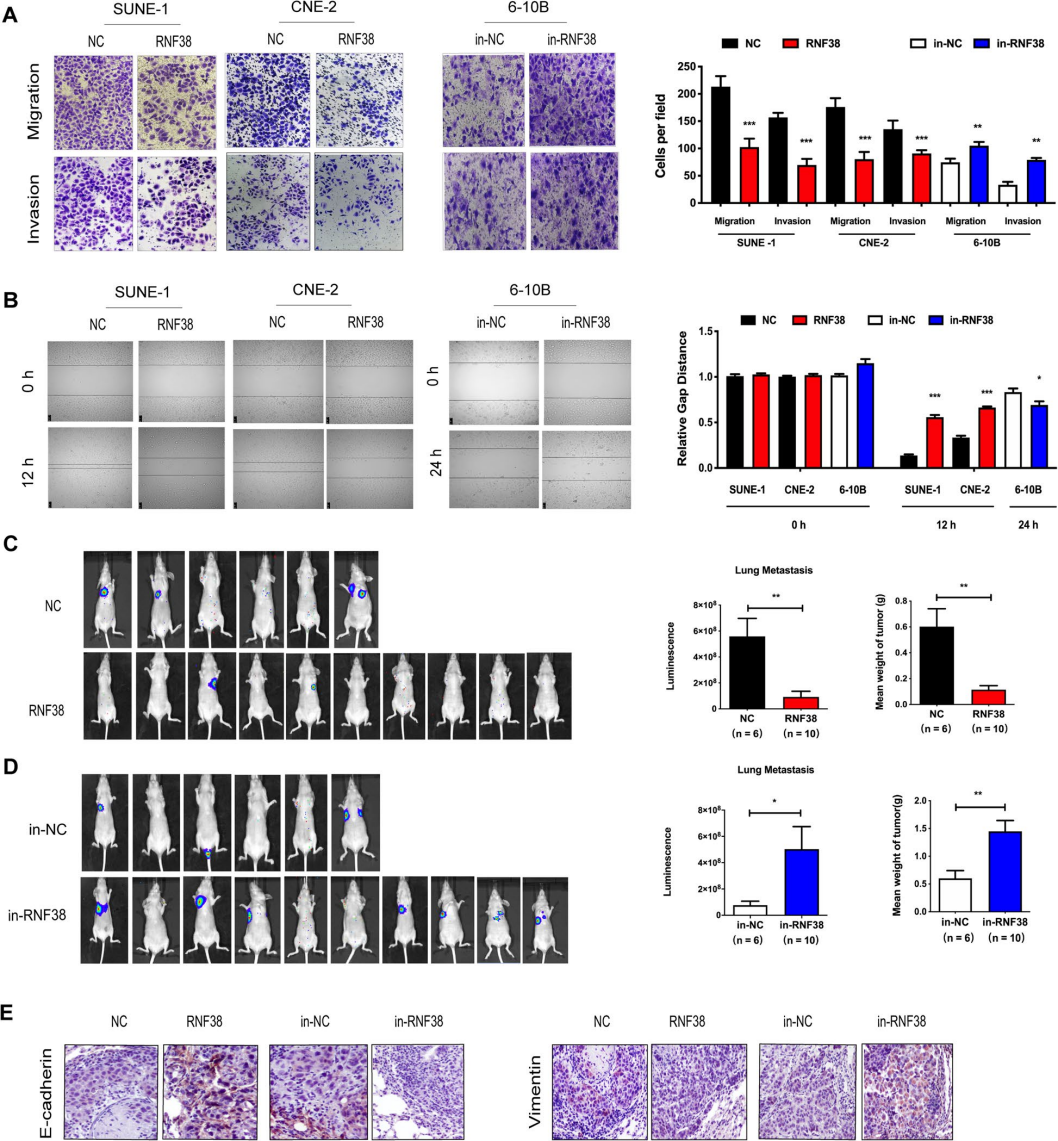
RNF38 inhibits NPC cell migration and invasion in vitro and in vivo. **A-B** Representative images and quantification of the migration and invasion assays in RNF38-upregulated (RNF38) SUNE-1 and CNE-2 cells and RNF38-downregulated (in-RNF38) 6-10B cells or their controls. **C-D** Intravenous injection of SUNE-1 cells stably overexpressing or downregulating RNF38 via the tail vein in the xenograft model (*n* = 6–10). Luminescence of lung metastasis and tumor weight were analysed. **E** Representative images of the expression of E-cadherin (Left column) and Vimentin (Right column) in xenograft tumors, *p < 0.05, ** *p* < 0.01

As RING finger E3s play a crucial role in cancer metastasis [28], we wondered whether RNF38 was involved in the process of NPC metastasis. Interestingly, we observed that compared with normal controls, RNF38 significantly attenuated NPC cell migration and invasion in SUNE-1 and CNE-2 cells (Fig. 4A-B). In contrast, downregulation of RNF38 remarkably enhanced the migration and invasion of 6-10B cells.

To further investigate whether RNF38 inhibits NPC metastasis in vivo, we injected stable control cells or RNF38-overexpressing SUNE-1 cells into nude mice through the tail vein. After 6 weeks, the mice were sacrificed, and the number of metastatic tumor nodules in the lungs was quantified. Notably, the data suggested that overexpression of RNF38 markedly inhibited the metastatic ability of SUNE-1 cells (Fig. 4C). The bioluminescence value and the average weight of metastatic lung lesions were remarkably higher in the RNF38-overexpressing nude mice than in the control mice. The opposite results were observed in the RNF38 downregulation group (Fig. 4D). In addition, we found lower E-cadherin and Vimentin expression in the RNF38-overexpressing group (Fig. 4E). Taken together, the above results showed that RNF38 served as a negative regulator of NPC metastasis.

### RNF38 suppresses NF-𝛋B and Erk1/2 pathways

**Fig. 5:**
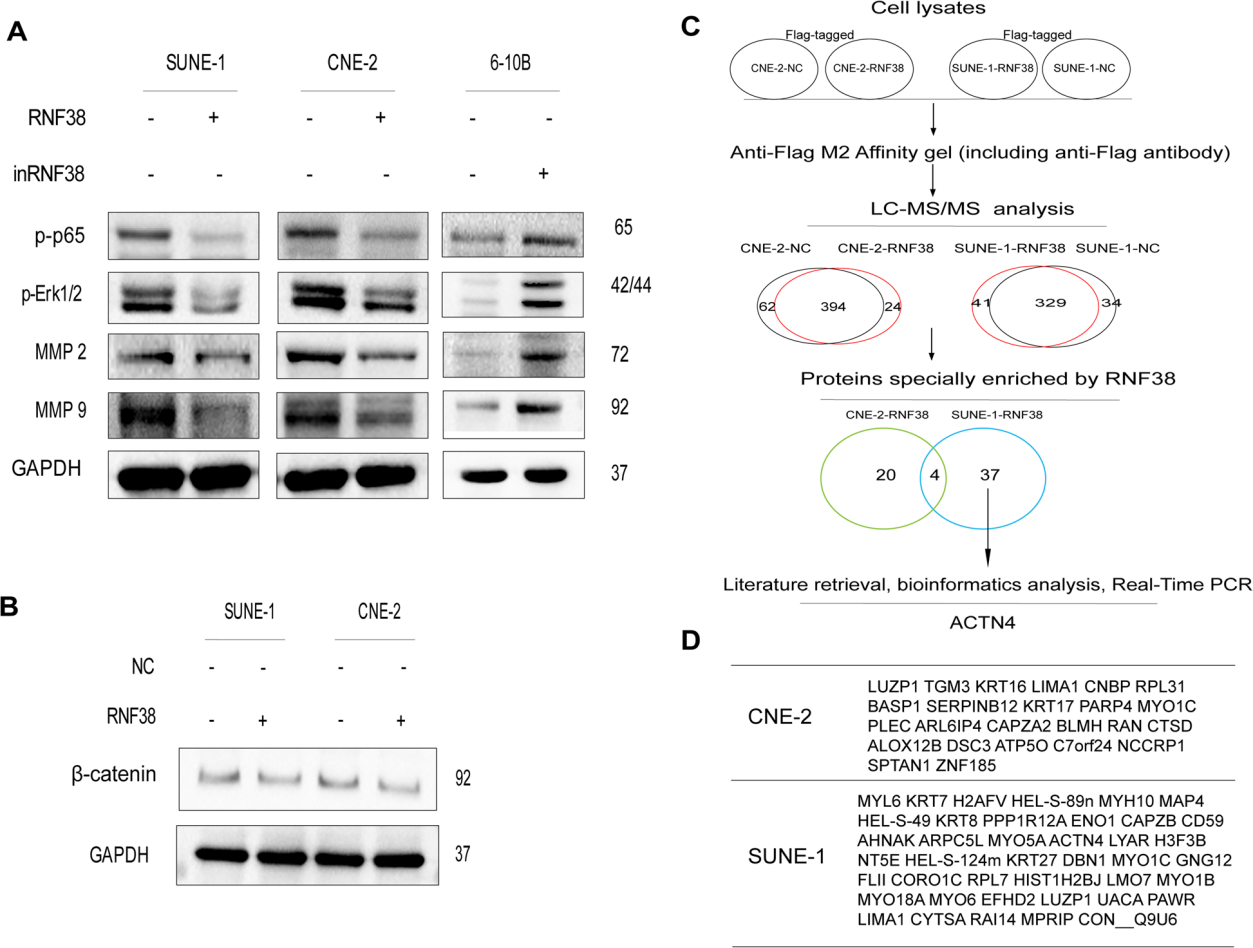
RNF38 inhibits activation of the NF-𝛋B and ERK1/2 signaling pathways. **A** Levels of p-p65, p-ERK1/2, MMP2 and MMP9 in RNF38-upregulated SUNE-1 and CNE-2 cells and in RNF38-downregulated 6-10B cells. **A** Levels of 𝛃-catenin in RNF38-upregulated SUNE-1 and CNE-2 cells. **C** Diagram of the identification processes of RNF38-interacting proteins. **D** Significant proteins enriched by CNE-2-RNF38 (*n* = 24) and SUNE-1-RNF38 (*n* = 41)

Then, we sought to explore the downstream signal transduction mechanisms by which RNF38 acts as a tumor suppressor in NPC. We found that upregulation of RNF38 inhibited the levels of p-p65 and p-Erk1/2 in RNF38-overexpressing SUNE-1 and CNE-2 cells (Fig. 5A). Conversely, downregulation of RNF38 reversed the protein changes in the NF-𝛋B and Erk1/2 signaling pathways. Of note, RNF38 had no effects on the WNT pathway (Fig. 5B). Furthermore, upregulation of RNF38 drove the expression of metastasis-related proteins, including MMP2 and MMP9 (Fig. 5A). Taken together, these data indicated that RNF38 inhibited growth and metastasis by suppressing NF-𝛋B and Erk1/2 signaling.

### Possible proteins interact with RNF38 according to liquid chromatography coupled with tandem mass spectrometry (LC-MS/MS)

To further investigate the deep mechanisms by which RNF38 influences the carcinogenesis of NPC, we overexpressed Flag-tagged RNF38 in NPC cells and performed a conventional pull-down with an anti-Flag antibody, and then performed LC-MS/MS to identify the possible proteins that interact with RNF38 (Fig. 5C). Eighty-six and 76 proteins were significantly enriched in CNE-2 cells and SUNE-1 cells, respectively. Compared to the control proteins, only 24 and 41 proteins were significantly enriched in the RNF38-overexpressing group in CNE-2 cells or SUNE-1 cells, respectively (Fig. 5D and Table S4). Functional analysis suggested that differentially expressed proteins were mainly enriched in the Gene Ontology (GO) terms actin cytoskeleton and focal adhesion in cellular component, actin binding, and cadherin binding in molecular function (Fig. S1A, Fig. S2A), which were closely associated with growth and metastasis in cancer. However, differentially expressed proteins were enriched in the Kyoto Encyclopedia of Genes and Genomes (KEGG) pathways of regulation of cytoskeleton in SUNE-1 cells (Fig. S1B) and apoptosis in CNE-2 cells (Fig. S2B) [29].

### RNF38 interacts with ACTN4 and induces ACTN4 degradation via ubiquitylation

**Fig. 6:**
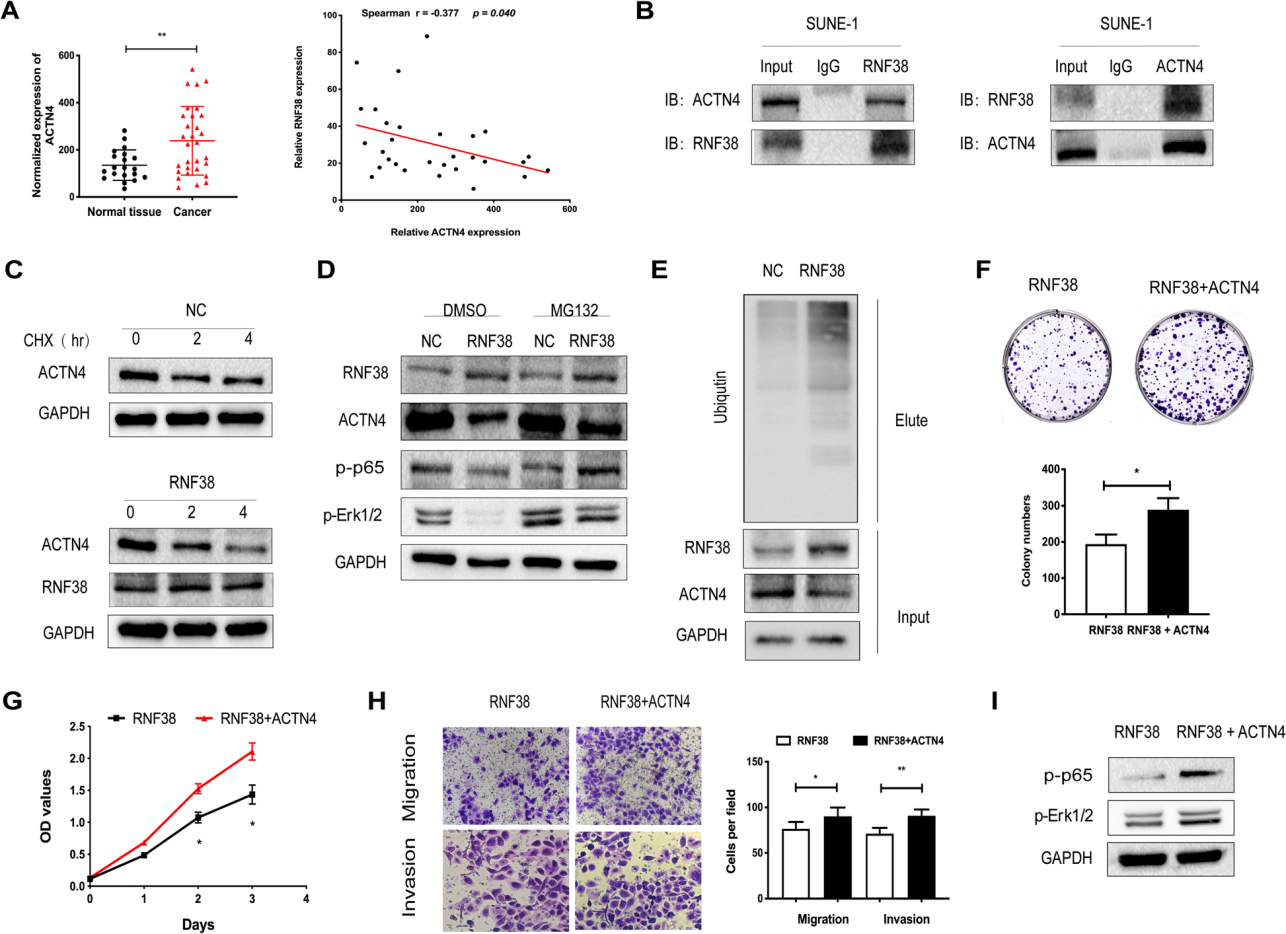
RNF38 interacts with ACTN4 and induces ubiquitylation and degradation of ACTN4. **A** ACTN4 expression in normal nasopharyngeal tissues and NPC tissues by PCR (Left column). Correlation of ACTN4 and RNF38 expression in nasopharyngeal carcinoma samples (Right column). **B** Coimmunoprecipitation detected the interaction of RNF38 and ACTN4 in SUNE-1 cells. **C** CHX chase assay was used to examine the half-life of ACTN4 in SUNE-1 cells treated with CHX for 0, 2 and 4 hours. **D** Western blotting was performed to observe the levels of RNF38, ACTN4, p-p65 and p-Erk1/2 in SUNE-1 cells treated with DMSO or MG132. **E** Immunoblotting using an anti-ubiquitin antibody was performed to examine the ubiquitination levels of ACTN4. F, Clone formation assays, proliferation assays (**G**), cell migration and invasion assays (**H**) in RNF38 overexpressing SUNE-1 cells with or without upregulation of ACTN4. I, Levels of p-p65 and p-Erk1/2 in overexpressing RNF38 SUNE-1 cells with or without upregulation of ATCN4

As a member of ubiquitin ligases (E3s), we hypothesize that RNF38 may suppress growth and metastasis through ubiquitination of related oncogene proteins in NPC cells. ACTN4, enriched in SUNE-1 cells, was chosen for further exploration by literature retrieval, bioinformatics analysis and Real-Time PCR. PCR showed that ACTN4 was significantly downregulated in NPC and negatively correlated with RNF38 (Fig. 6A). Co-immunoprecipitation (Co-IP) experiments in RNF38-overexpressing SUNE-1 cells showed that RNF38 binds to ACTN4, and reciprocal Co-IP further confirmed the interaction between RNF38 and ACTN4 using endogenous ACTN4 and RNF38 antibodies (Fig. 6B). The CHX assay suggested that the upregulation of RNF38 shortened the half-life of the ACTN4 protein (Fig. 6C). In addition, upregulation of RNF38 decreased the levels of ACTN4, while the expression of ACTN4 was restored when treated with MG132 (Fig. 6D). Moreover, suppression of NF-𝛋B and Erk1/2 signals was somewhat attenuated when cells were treated with MG132. In addition, immunoblotting using an anti-ubiquitin antibody indicated that overexpression of RNF38 promoted ACTN4 ubiquitination (Fig. 6E). Together, these results imply that ACTN4 interacts with RNF38 and that RNF38 promotes ACTN4 ubiquitination and degradation.

To further confirm the impacts of ACTN4 in the tumor-inhibiting effects of RNF38, rescued ACTN4 expression in growth and invasion assays are conducted. Our data showed that upregulation of ACTN4 facilitated NPC cell growth (Fig. 6F-G) and metastasis (Fig. 6H) compared with RNF38 overexpressing SUNE-1 cells. Moreover, ACTN4 reversed NF-𝛋B and ERK1/2 pathways by overexpressing ATCN4 together with RNF38 (Fig. 6I). Collectively, RNF38 suppress the development of NPC by interacting with ACTN4.

## Discussion

Despite the great success of IMRT and concomitant chemoradiotherapy in NPC, the recurrence and metastasis rate has remained high (15–30%) over the past few decades [5]. Our study found that RNF38 was downregulated in NPC cells and clinical samples. Lower expression of RNF38 in primary NPC was an independent unfavorable prognostic biomarker of DMFS and OS. Importantly, our data suggested for the first time that RNF38 was a tumor suppressor gene in NPC. Overexpression of RNF38 inhibited cell proliferation, migration, and invasion in vitro and lung metastasis in a xenograft mouse model. Mechanistic studies revealed that RNF38 suppressed growth and metastasis by attenuating the NF-𝛋B and ERK1/2 signaling pathways via ubiquitination and degradation of ACTN4. RNF38 inhibits the development of NPC by interacting with ACTN4.

Notably, RNF proteins play important roles in both tumor suppression and oncogenesis [28]. RNF43 attenuated tumor growth and the stem cell-like phenotype by inhibiting the canonical Wnt/β-catenin pathway in gastric cancer, and loss of RNF43 was closely associated with poorer prognosis [10]. The silencing of RNF20 contributed to inflammation-associated colorectal cancer by activating the NF-𝛋B pathway [11]. In addition, RNF38 significantly inhibited the growth of cervical carcinoma cells [30]. However, compared with the above three RNFs, RNF125, RNF6, and RNF183 have the opposite effects in cancers. RNF125 was found to promote invasion and metastasis of gallbladder cancer via the TGF-β1-SMAD3-ID1 pathway and was associated with worse outcomes [12]. RNF6 was upregulated in colorectal cancer [13, 31], breast cancer [14], leukemia [32], and prostate cancer [33]. RNF6 amplification could activate the JAK/STAT3 or Wnt/β-catenin pathway and serve as an unfavorable prognostic marker in colorectal cancer. Similarly, RNF183 also contributes to the carcinogenesis of colorectal cancer, similar to RNF6 [15]. However, as a family member of the RING finger family, the role of RNF38 in NPC remains unknown. Our study suggested that, like RNF43 and RNF20, RNF38 plays a tumor-suppressing role in NPC and is supposed to be a potential biomarker for predicting recurrence and metastasis according to our study. High expression of RNF38 was significantly associated with prolonged OS and DMFS but not LRFS and RRFS. Previous studies suggested that RNF38 promoted the progression of hepatocellular carcinoma, gastric cancer, and non-small-cell lung cancer [34–36]. This inconsistency might be linked to the diverse biological behaviors in different cancers, which need to be addressed in future studies. Therefore, the role of RNF38 remains to be validated in more cancers.

A previous study indicated that RNF expression could affect cancer cell sensitivity to DNA-damaging drugs [15]. We also found that instead of affecting NPC cell sensitivity to cisplatin, RNF38 promoted apoptosis of NPC cells when treated with etoposide. It seems possible that etoposide was a cell cycle-specific agent and principally killed cells in the S phase, while cisplatin was a cell cycle nonspecific agent. Therefore, overexpression of RNF38 facilitated apoptosis of NPC cells to etoposide.

To investigate the mechanisms underlying RNF38-driven inhibition in NPC, the NF-𝛋B pathway and MAPK pathway were further examined, which were proven to be the major altered signaling pathways in NPC [37, 38]. It is worth noting that NF-𝛋B and MAPK pathway aberrations accounted for 34.2 and 11.7% in NPC, respectively [39]. Our study indicated that RNF38 exhibited a negative effect on growth and metastasis in NPC by suppressing NF-𝛋B and MAPK activation instead of activating the NF-𝛋B pathway by RNF20 or RNF183 in colorectal cancer [11, 15]. Of note, as a member of the UPS, whether RNF38 ubiquitination plays a role in NPC remains unclear. Previous studies showed that RNF20 was associated with histone H2B ubiquitination in inflammation-related colorectal cancer [11]. RNF6 was reported to promote colorectal cancer by ubiquitination of TLE1 in prostate cancer [40]. With the help of LC-MS/MS, we determined the proteins significantly enriched by RNF38. Our study further found that RNF38 could also function through ubiquitination of ACTN4 in NPC cells.

## Conclusions

Our study indicated that RNF38 functions as a tumor suppressor. RNF38 inhibits growth and metastasis by inducing ubiquitination and degradation of ACTN4 in NPC cells. RNF38 serves as an independent biomarker in NPC patients, which provides a novel strategy to guide clinical practice.

## Supplementary Information

**Additional file 1: Figure S1.** Gene Ontology (GO) analysis (**A**) and Kyoto Encyclopedia of Genes and Genomes (KEGG) analysis (**B**) of significantly enriched proteins in RNF38-overexpressing SUNE-1 cells.

**Additional file 2: Figure S2.** Gene Ontology (GO) analysis (**A**) and Kyoto Encyclopedia of Genes and Genomes (KEGG) analysis (**B**) of significantly enriched proteins in RNF38-overexpressing CNE-2 cells.

**Additional file 3: Supplementary Table S1.** Comparison of RNF38 expression between nasopharyngeal carcinoma and normal nasopharyngeal epithelia tissues.

**Additional file 4: Supplementary Table S2.** Immunohistochemistry score of RNF38 expression in nasopharyngeal carcinoma and normal nasopharyngeal epithelia tissues.

**Additional file 5: Supplementary Table S3.** Characteristics of patients with nasopharyngeal carcinoma grouped by RNF38 expression.

**Additional file 6: Supplementary Table S4.** Proteins that specially enriched by RNF38 in SUNE-1 and CEN-2 cells. (XLS 67 kb)

## Data Availability

All data generated or analysed during this study are included in this published article and its supplementary information files.

## Abbreviations

NPC: Nasopharyngeal carcinoma
RNF: RING finger proteins
IMRT: Intensity-modulated radiation therapy
Es: Enzymes
IHC: Immunohistochemistry
BART: BamHI-A rightward transcripts
DMFS: distant metastasis-free survival
LRFS: Local relapse-free survival
RRFS: Regional relapse-free survival
LC-MS/MS: Liquid chromatography coupled with tandem mass spectrometry
OS: overall survival
GO: Gene ontology
KEEP: Kyoto Encyclopedia of Genes
UPS: ubiquitin-proteasome system
