# Supplementary figures and images for: RNF38 suppress growth and metastasis via ubiquitination of ACTN4 in nasopharyngeal carcinoma
Source: BMC Cancer. 2022 May 15;22:549. doi: 10.1186/s12885-022-09641-x (PMC9107765; doi:10.1186/s12885-022-09641-x)

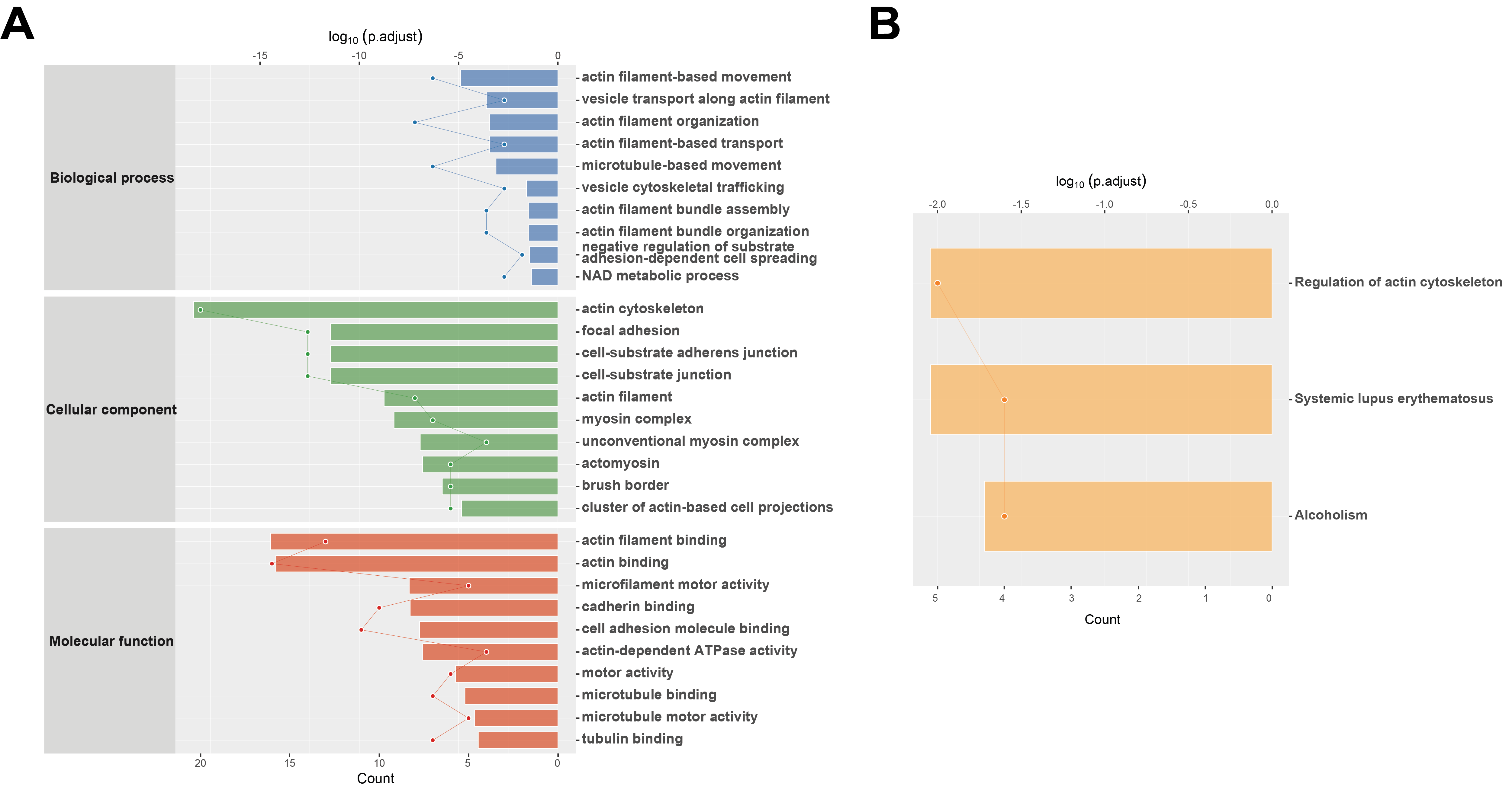

Supplement: Supplementary file 1 — Additional file 1: Figure S1. Gene Ontology (GO) analysis (A) and Kyoto Encyclopedia of Genes and Genomes (KEGG) analysis (B) of significantly enriched proteins in RNF38-overexpressing SUNE-1 cells. [file 12885_2022_9641_MOESM1_ESM.png]

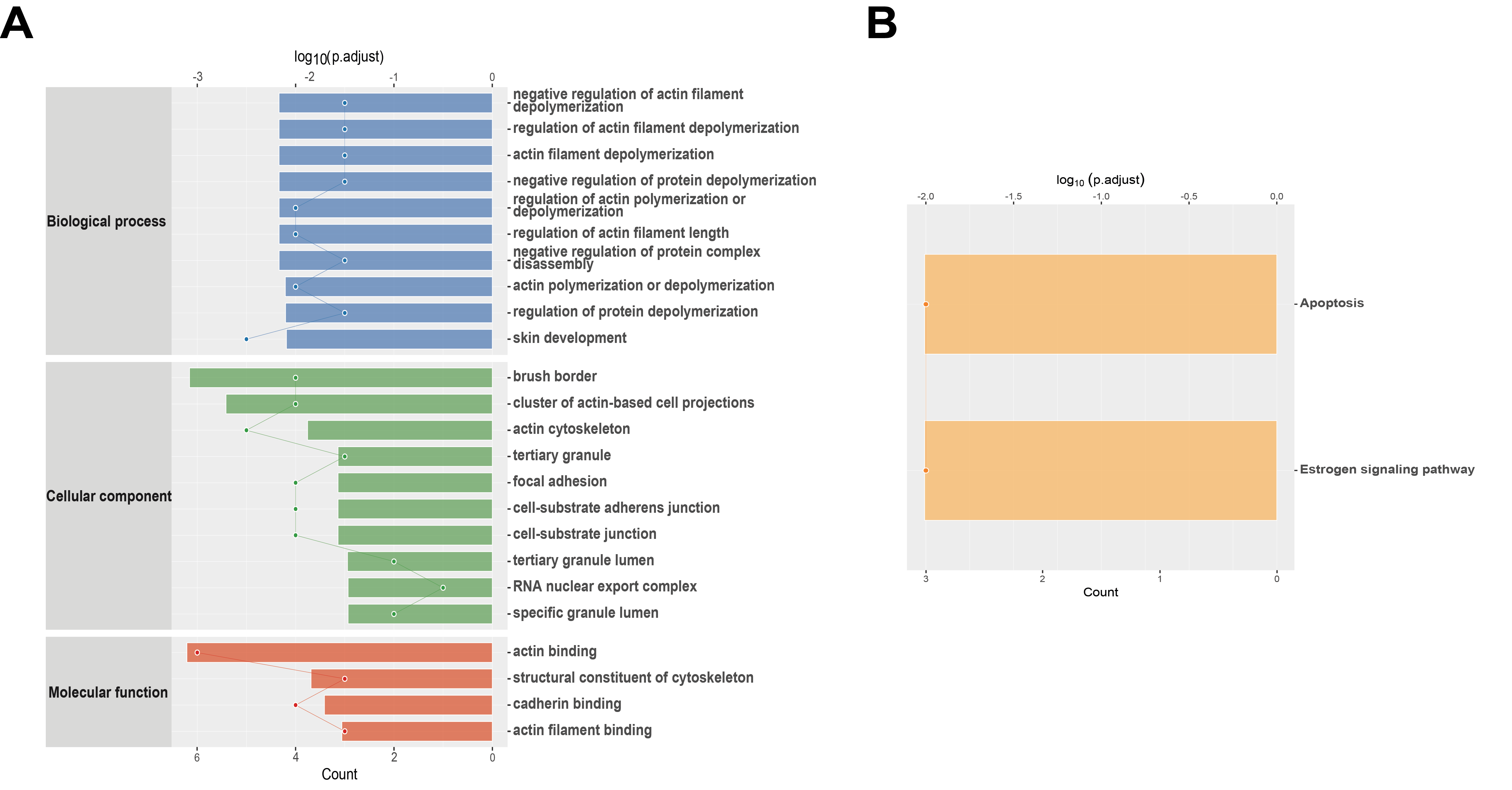

Supplement: Supplementary file 2 — Additional file 2: Figure S2. Gene Ontology (GO) analysis (A) and Kyoto Encyclopedia of Genes and Genomes (KEGG) analysis (B) of significantly enriched proteins in RNF38-overexpressing CNE-2 cells. [file 12885_2022_9641_MOESM2_ESM.png]
