# Supplementary material for: RNF38 suppress growth and metastasis via ubiquitination of ACTN4 in nasopharyngeal carcinoma
Source: BMC Cancer. 2022 May 15;22:549. doi: 10.1186/s12885-022-09641-x (PMC9107765; doi:10.1186/s12885-022-09641-x)
Supplement: Supplementary file 3 — Additional file 3: Supplementary Table S1. Comparison of RNF38 expression between nasopharyngeal carcinoma and normal nasopharyngeal epithelia tissues. [file 12885_2022_9641_MOESM3_ESM.doc]

**Supplement Table S1** Comparison of RNF38 expression between nasopharyngeal carcinoma and normal nasopharyngeal epithelia issues

| Groups | RNF38 | | *p* |
| --- | --- | --- | --- |
|  | Positive | Negative |  |
| Nasopharyngeal carcinoma | 54 | 75 | 0.0016 |
| Normal nasopharyngeal epithelia | 16 | 4 |  |
