# Supplementary material for: RNF38 suppress growth and metastasis via ubiquitination of ACTN4 in nasopharyngeal carcinoma
Source: BMC Cancer. 2022 May 15;22:549. doi: 10.1186/s12885-022-09641-x (PMC9107765; doi:10.1186/s12885-022-09641-x)
Supplement: Supplementary file 5 — Additional file 5: Supplementary Table S3. Characteristics of patients with nasopharyngeal carcinoma grouped by RNF38 expression. [file 12885_2022_9641_MOESM5_ESM.doc]

**Supplement Table S3** Characteristics of patients with nasopharyngeal carcinoma grouped by RNF38 expression

| Variables |  | RNF38 expression | | | *p* |
| --- | --- | --- | --- | --- | --- |
|  | Overall（n=129） | Negative（n=75） | Positive（n=54） | |  |
| sex |  |  |  | 0.296 | |
| Male | 78 | 45 | 33 |  | |
| Female | 51 | 30 | 21 |  | |
| Age at diagnosis |  |  |  | 1.000 | |
| ≤ 50 years | 106 | 58 | 48 |  | |
| > 50 years | 23 | 17 | 6 |  | |
| T classification |  |  |  | 0.096 | |
| T1-2 | 48 | 23 | 25 |  | |
| T3-4 | 81 | 52 | 29 |  | |
| N classification |  |  |  |  | |
| N0-1 | 89 | 52 | 37 | 1.000 | |
| N2-3 | 40 | 23 | 17 |  | |
| AJCC stage |  |  |  | 0.223 | |
| I-II | 33 | 16 | 17 |  | |
| III-IV | 96 | 59 | 37 |  | |
| Chemotherapy |  |  |  | 1.000 | |
| No | 18 | 11 | 7 |  | |
| Yes | 111 | 64 | 47 |  | |
